# Decision Tree of Materials: A Model of Halal Control Point (HCP) Identification in Small-Scale Bakery to Support Halal Certification

**DOI:** 10.1155/2022/5244586

**Published:** 2022-04-12

**Authors:** Sucipto Sucipto, Reza Wahyu Damayanti, Claudia Gadizza Perdani, Muhammad Arif Kamal, Retno Astuti, Nur Hasanah

**Affiliations:** ^1^Halal-Qualified Industry Development (Hal-Q ID), Universitas Brawijaya, Malang 65145, Indonesia; ^2^Department of Agroindustrial Technology, Faculty of Agricultural Technology, Universitas Brawijaya, Malang 65145, Indonesia; ^3^Department of Psychology, Faculty of Social and Political Sciences, Universitas Brawijaya, Malang 65145, Indonesia

## Abstract

A bakery is a business that bakes flour-based foods, including bread, cookies, cakes, pastries, and pies, and sells them. Some bakeries are also categorized as large scale, medium scale, and small scale. Halal embraces all food category; bakery product can satisfy the challenge and opportunity of halal food segment market. The small-scale bakery will benefit from creating a halal certification to attract new customers. The first stage of submission for halal certification is identifying Halal Control Points (HCP) of materials and production. The material tracing uses a decision tree. The purpose of this study is to identify HCP in materials and production processes and provide alternative improvements. Identification of HCP in material decision trees to determine contains non-HCP (halal) material, HCP material, and haram (forbidden) material. Happy Cake bakery uses 75% non-HCP (halal) materials and 25% HCP (noncertified halal) materials from 80 ingredients. Bakery Canggi Fully has 83.3% halal materials and 16.6% noncertified halal materials from 24 ingredients. Bakery MacCheese has 79% of halal materials and 21% of noncertified halal materials from 43 ingredients. The decision tree makes it very easy to identify the halal status of ingredients. The HCP ingredients need to be replaced with clearly halal ingredients. Substitution of HCP material to halal-certified ingredients may affect production costs, product quality, and profit. Therefore, it is necessary to choose a suitable halal material. Halal certification requires a high commitment of small-scale bakery businesses.

## 1. Introduction

Indonesia is one of the largest Muslim populations in the world. Data from Global Islamic Economic Report 2018-2019, Indonesia's Muslim population reaches 87.18% from 232.5 million or 202,693,500. As a Muslim majority country, halal is the main parameter in choosing and consuming products. Law No. 33 of 2014 Regarding Halal Product Guarantee (HPG) on article 4 states that products entering, distribution, and trading in Indonesia must be halal certified [1]. The demand for halal products continues to increase [2]. One of the people's demands and choices is the bakery. A bakery is a food group made from wheat flour, consumed as a staple food, snacks, and dessert. Bakery products have a variety of shapes, attractive, and varied flavors. Bakery products are following the trend [3]. The bakery can last a week, tastes good, is easy to store, is easy to obtain, and does not require preparation time to consume [4]. This condition makes bakery products preferred by consumers who have a dynamic lifestyle. Bakery product has captivated consumers because of its nutrition and possibility food aid during disasters [5]. According to the Center For Agricultural Data and Information System [6], the average consumption of processed flour, such as bread, increased from 2009 to 2015 by 2,586 kg per capita. Bakery producers are not only from large and medium industries but also small-scale businesses. The number of small-scale bakery businesses in Indonesia is vast. Many small-scale bakeries are not yet halal-certified. Several researchers have conducted similar studies on hygienic, safety, and quality aspects in developing small-scale bakeries. These researches include: hygiene rules in the bakery industry [7], food safety problems in developing countries [8], Good Manufacturing Practices (GMP) and Sanitation Standard Operating Procedure (SSOP) evaluation in bakery Small Medium Enterprises (SMEs) [9], improving the quality of the baking industry [10].

On the other hand, research on halal certification development for small enterprises, especially bakeries, is still limited. Evaluation of Halal Assurance System (HAS) in small and medium industry bakery is limited to one business [11]. However, halal certification affects consumer purchases. Halal logo's existence affects tempeh chip purchasing [12]. Halal food supports halal tourism [13]. Part of the halal certification stages is document formulation and implementation of the Halal Assurance System (HAS). The initial step is identifying the halal control point (HCP) of the material and the production process. The guideline for HCP identification is used to prepare the HAS document. HAS 23101 criteria for the processing industry consist of 11 principles, namely, halal policy, halal management team, training and education, materials, products, production facilities, written procedures for critical activities, traceability, a process for handling products that do not meet criteria, internal audit, and review remanagement. Bakery quality is influenced by fat, water incorporation, heat transfer, tenderness and texture, moisture, mouthfeel, flavor, and shelf life [14–16]. Bakery ingredients are diverse and very complex to form dough and bakery products with good sensory. Fat can be from vegetable fats or a mixture of vegetable and animal fats. Even biscuits can use a source of fat from butter, sunflower oil, or lard [3]. Production of good quality bakery needs to choose the main ingredients (major) and additional ingredients (minor). The biscuit's major ingredients are flour, fat, sugar, water, and chemical leavening agents (sodium bicarbonate and ammonium bicarbonate). Salt, egg, emulsifier, milk powder, and flavoring compounds are minor ingredients [17]. The three major ingredients of cookies are flour, sugar, and fat. The types of cookies depend on the composition, dough making, and baking parameters [18]. These materials are produced by various companies using a variety of materials and methods. Therefore, tracing the halal status and the possibility of prohibition materials are essential in these bakery industries. For example, some cheese is coagulation of milk using a starter or/and enzymes from microbial, vegetable, and in case of animals' digestive tract. If the enzyme from an animal's digestive tract is haram, the cheese has the status of haram. Tracing HCP bakery small-scale with various products and ingredients is not easy. Some of the raw materials that need to be investigated for their halalness are wheat flour, developer materials, meat, and processed products, rhum, emulsifier, shortening, margarine, yeast, cheese, creamer, chocolate, gelatin, food coloring, and flavoring [19]. In addition to recognizing product characteristics, one must also understand the components and composition of the ingredients. The decision tree helps to trace the elements that include HCP in the material. The purpose of this study was to identify HCP in materials and small-scale bakery production and provide alternative improvements to support the halal certification submission.

## 2. Materials and Methods

### 2.1. Research Object

This research was conducted in three small-scale bakery businesses, i.e., Happy Cake, Canggi Fully, and MacCheese, in Malang City, East Java, Indonesia. The respondents consisted of ten people, four of whom belonged to Happy Cake, three from Canggi Fully, and three from MacCheese. The respondents consist of owners and production employees of each company. The products identified in the Happy Cake bakery are 28 composed of 80 ingredients. Bakery Canggi Fully has nine products using 24 ingredients. Bakery MacCheese has eight products using 43 ingredients.

### 2.2. Research Procedure

HCP determination is based on the HAS 23101 critical activity procedure in the processing industry. Critical activity procedures include selecting new materials, purchasing materials, inspecting incoming materials, formulating products/development of new products, an inspection of incoming materials, production, washing production facilities and auxiliary equipment, storage, and handling materials and products along with transportation. The study begins with analyzing the business's general condition, identifying the product types and ingredients, fulfilling the criteria for critical activity procedures, and handling materials that do not meet the criteria. The status of the halal material was traced using a decision tree. Materials that do not meet the requirements are given alternative halal materials to ensure that the final product is halal. The final stage is the implications of replacing materials for production costs, product quality, and profits generated.

### 2.3. Research Types

This research uses descriptive qualitative and quantitative approaches. The qualitative descriptive analysis explains qualitative data, while quantitative descriptive analysis explains quantitative data using the tabulation method [20]. Descriptive qualitative methods provide an overview of the business, product descriptions, and procedures for critical activities. In contrast, the quantitative analysis method was used for calculating the questionnaire results of the internal audit.

## 3. Results and Discussion

### 3.1. General Description of Bakery Business

#### 3.1.1. Happy Cake Bakery

Happy Cake is one of the small-scale bakeries established in 2007 in Malang, East Java. This business produces 19 types of bakery, from bread to cookies. In this business, there are six workers, consisting of 3 production workers, one packaging person, one marketing person, and one courier. The online marketing of Happy Cake is promoting through WhatsApp and Instagram. Happy Cake's turnover reaches IDR 27 million/month.

#### 3.1.2. Canggi Fully

Canggi Fully is a small business that produces bakery types of pastries and pastries. Canggi Fully was established in 2016 with an initial capital of IDR 500,000. This business has two employees in the production department. Canggi Fully marketing is online and offline. Online marketing is via WhatsApp and Instagram, while offline marketing opens booths in front of the house and Car Free Day (CFD) on Jalan Idjen every Sunday. Sales turnover is IDR 10 million/month.

#### 3.1.3. MacCheese

MacCheese is one of the bakery businesses in Malang and was established in 2014, producing food and beverages, namely, ice mochi, bakery, and pasta. The number of workers is three people consisting of 2 people in production and 1 in packaging. Online marketing is promoting through WhatsApp and Instagram. Turnover is IDR 20 million/month.

### 3.2. Product Description

The product description contains information about the product types. Happy Cake bakery business has superior croissant products, Canggi Fully moringa crackers, while MacCheese banana Bolen. A list of products for each bakery business can be seen in Table 1.

### 3.3. Written Procedure for Critical Activity

#### 3.3.1. Material Selection

Material is selected for approval of its use. The material selection procedure must guarantee that every ingredient used is approved by the Halal Certification Body. HCP identification of each ingredient is based on a decision tree. The halal decision tree is used as a reference to replace unclear halal material with clearly halal materials.

#### 3.3.2. Mapping of HCP Decision of Animal Materials

**Figure 1:**
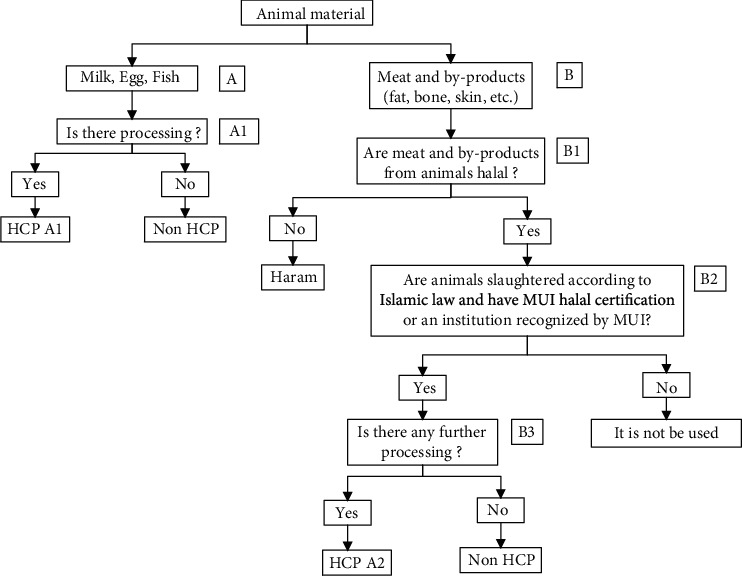
Decision tree for identification of Halal Control Points for animal materials (source: [21]). The explanation is as follows: A: list of questions for animal ingredients, milk, eggs, and fish; B: list of questions for animal ingredients and by-products (fat, bones, skin, etc.); number: question sequence.

Animal material is one or several parts of the animal's body. Animal ingredients are identified, starting from fresh ingredients to processed ingredients. The identification of animal material halal status based on the decision tree refers to Figure 1.

Slaughter must meet Islamic law and already have an official halal certificate. If there is further processing, it must be ensured to use halal certified materials to guarantee their halal statute. HCP of ingredients, three bakeries were identified through a question tree-based decision tree in Table 2.

Beef floss is an HCP A2 material with nonhalal potential, namely, the slaughter process and monosodium glutamate (MSG) material. The method of slaughtering can cause halal or haram animal material if it is not under Islamic law. MSG is a reaction product from glutamic acid, and NaOH is produced involving microbes, so it needs to be observed by microbial growth media for fermentation and criticism of halal status. Milk powder, whip cream, butter, and cheese are materials HCP A1 material with the potential of nonhalal ingredients, namely, lactose (milk sugar), stabilizer, emulsifier, etc. The stabilizer maintains the viscosity of the milk powder [22]. The stabilizer is usually an emulsifier. In general, the emulsifier status is doubtful because it can be made from vegetable or animal material [23]. Shrimp for the bakery business is the raw material (not processed) so that it is classified as Non-HCP (A) or without any potential for nonhalal content. Whip cream is an HCP A1 material with the potential for nonhalal ingredients, namely, milk, emulsifier, and flavor. Most emulsifiers are derivatives of fat, so they need to be observed halal. Whip cream is usually sweetened and mixed with vanilla flavor to add flavor [24]. Butter is an HCP A1 material with the potential of nonhalal ingredients, namely, milk, animal fat, and emulsifier. Butter is a combination of milk and fat to form solids. The milk used is fresh milk or fermented milk, while animal fat from nonhalal animals such as pigs becomes haram (forbidden) [24]. Also, making butter is often added to the emulsifier to increase stability. Eggs include Non-HCP animal ingredients (A), so there is no haram potential. Eggs are halal [25]. Chicken meat is an animal raw material through slaughter. Often chicken meat is considered halal because it is a halal animal. Chicken meat needs to be slaughtered according to Islamic teachings to ensure its halalness. The process of slaughtering can make halal or haram chicken meat so that HCP A2 is categorized. The halalness of chicken meat depends on the process, slaughtering equipment, and distribution. Noerdyah et al. have discussed the risk mitigation of halal and safety on the broiler [26], Sucipto et al. have presented the risk of beef production, and Hidayati et al. have researched the sustainability aspects of chicken slaughter [28]. Poultry slaughtering must produce Aman, Sehat Utuh, dan Halal (ASUH), or safe, healthy, halal, whole chicken products. A universal halal standard does not exist [29]. Some slaughtering was with and without stunning. Halal slaughter without stunning is legal [30–32]. Despite this, stunning is accepted if it does not cause death before being slaughtered [33]. In Indonesia, the regulation of halal slaughter on poultry and ruminants follows the Indonesian National Standard (SNI) and the fatwa of the Indonesian Ulema Council [34–36].

Some cheese are fermented processed ingredient from milk, so it includes HCP A1 material whose potential of nonhalal elements is an enzyme to agglomerate milk, emulsifier, color, and flavor. Cheese resulted from the ripening and clumping of milk using rennet and acid enzymes [37]. Cheese, including microbial products, need lactobacillus for fermentation, so it must be ensured that the microbial growth media is halal. The addition of emulsifiers and flavor in cheese processing is not yet clearly halal [24].

Flavors can be produced from plants, animals, and synthetic. Flavoring that is made from animal ingredients includes HCP A2. The potential for nonhalal ingredients is monosodium glutamate (MSG). MSG is often added for savory taste in products [38]. Synthetic flavoring is often made from fatty materials and/or amino acids from animals through the Maillard reaction [39]. MSG is the sodium salt of glutamic acid from glutamic acid and NaOH reaction [24]. Commercially, glutamic acid is produced using microbes, so it needs to be criticized halal. Therefore, the halalness of flavoring must be traced.

#### 3.3.3. Mapping of Decision Tree HCP of Vegetable Material

**Figure 2:**
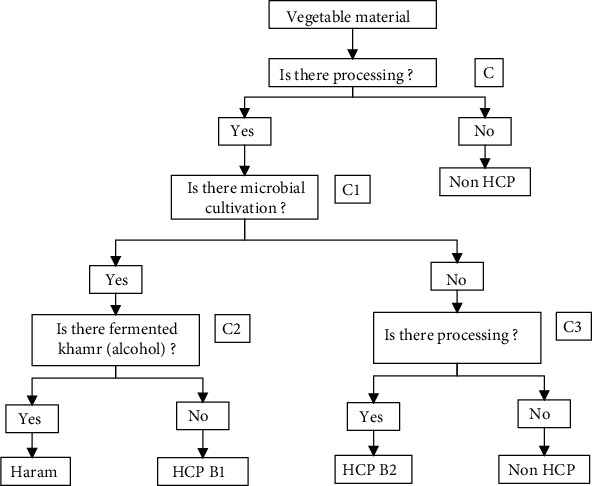
Decision tree for identification of Halal Control Points for vegetable materials (Source: [21]). The explanation is as follows: c: list of questions for vegetable materials; number: question sequence.

Vegetable material is obtained from plants. Vegetable ingredients do not necessarily only contain fresh vegetable material. They usually processed vegetable ingredients plus food additives to improve quality. Vegetable food ingredients need to be identified its HCP, which is material from plants, whether fresh or processed. The identification of the halal status of vegetable material is based on the decision tree in Figure 2.

Vegetable material without processing becomes Non-HCP (halal), for example, fresh pineapple and potato. Processed vegetable ingredients using khamr (alcohol) fermentation is including illegitimate products. Fermentation without khamr must be known for ingredients used to ensure halal. The HCP of all materials to produce bakeries must be identified through the decision tree (Table 3).

Vegetable ingredients such as margarine, strawberry jam, and wheat flour are HCP B2 ingredients because of processing and food additives. Margarine is butter from vegetable fat. Margarine is often added with antioxidants to prevent rancidity, flavor to have a fresh aroma, and food coloring to make it more attractive [24]. Also, margarine plus an emulsifier reduces the splashing of the product when used for frying. Jam has the potential for nonhalal ingredients, namely, flavor, sugar, preservatives, and coloring. Jam production is often added to preservatives to extend the shelf life. Jam production was added citric acid and thickening substances such as pectin. Citric acid is a microbial product that needs to be observed in microbial growth media for fermentation [40].

Wheat flour processing is often added to vitamins and L-cysteine. Wheat flour from wheat seeds is often enriched with additives such as vitamin A [37]. Vitamin A is usually coated to dissolve quickly and is not easily damaged during storage [41]. Coating materials other than halal materials such as gum are also from syubhat (dubious) materials such as gelatin. L-Cysteine is an improving agent to improve the properties, and quality of the flour [42] stated that L-cysteine could be made from human hair and animal hair. If L-cystein is made from human hair, it is including non halal material. L-Cysteine from animal hair must be ascertained from halal animals.

Chocolate powder is an ingredient of HCP B2 because there are processing and adding sugar, milk, emulsifier, and lecithin. Lecithin is abundant in soybeans, egg yolks, and animals' brains, so it needs to be observed from halal or haram animals [37]. The halal animal slaughter meets Islamic law or has been certified halal. Refined sugar includes HCP B2 because processing product needs to be checked. Refined sugar is refined sugar (sucrose), so HCP depends on sucrose production. Sucrose, at one stage of sugarcane production, is produced through bleaching. Bleaching sometimes uses activated carbon. Not all activated carbon is from plants, but there are from the bones of animals such as cows and pigs [24]. Active charcoal from animal bones is rarely found, and many active charcoals are from plants [42].

#### 3.3.4. HCP Decision Tree Mapping Microbial Material

Microbes are widely used to process various foods into products. LPPOM MUI has issued a fatwa regarding microbial materials [43]. The microbial materials that need grow media, additive materials do not come from pigs and their derivatives and do not harm human health are permitted. However, identification of the halalness of all microbial material is including HCP.

**Figure 3:**
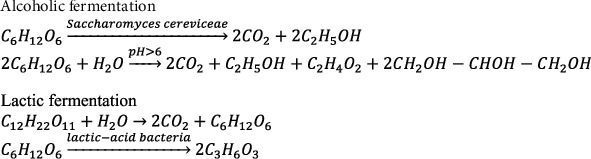
Sugar metabolism in the dough. Modified after [44, 45].

In this study, microbial materials were used by the Happy Cake and MacCheese bakery businesses with yeast brands Instant saf, Mauripan, and Fermipan. Bread yeast is a biomass/cell Saccharomyces cerevisiae fermented on carbon and nitrogen media [37]. The use of yeast in the dough is essential because it determines the proofing of the bakery. This fermentation process occurs metabolism of sugar in the dough, as shown in Figure 3. The fermented CO_2_ determines the development of the matrix during baking [44].

Generally, making yeast is added antifoam to prevent the formation of too much foam. Yeast is sometimes modified by adding emulsifiers to increase the dough's development capacity [24]. Fermentation in bread is to develop in bread dough, not to produce ethanol. The HCP of halal bioproducts includes fermentation products: (i) microorganisms' source, (ii) growth medium, (iii) fermentation, (iv) downstream processing, and (v) packaging and labeling [46, 47]. Fermentation time to form bread dough is less than 12 hours. Bread with a high alcohol content has yet to be found [48]. The bread dough made by fermentation can produce 0.3-0.4% alcohol, not a haram problem. The presence of alcohol after baking is not a concern [49].

#### 3.3.5. Purchasing Materials

Material purchasing procedure refers to the list of halal materials that the Halal Certification Body. Purchasing must have supporting data such as the name of the material, code, company name, factory location, and halal logo. Suppose there is a material that has not been certified as halal. In that case, it must be replaced with halal-certified material or have a halal production process statement from the producer that needs to be verified by the halal inspector. An example of material purchase information is shown in Table 4.

#### 3.3.6. Inspection of Incoming Materials

When the incoming material is checked, the physical condition, including freshness, shape, color, expired date, brand name, conformity of the order with acceptance, and halal status. The material inspection ensures that the label's information matches the material supporting documents (material name, producer name, country of origin, and halal logo). If the package's information is incomplete, the company must request supporting documents to ensure its halal status.

#### 3.3.7. Product Formulation and New Product Development

All bakery products of Happy Cake, Canggi Fully, and MacCheese have detailed ingredients formulation for production. Formulations determined by the business owner must be the basis of the production to maintain quality and halal products. If it does not match the formula, the halal product is not guaranteed. In addition, the production process that is not good and standardized will cause the quality of the product to become less good, like bread is burnt, too hard, and does not expand. There are no new product developments in the three bakery businesses. Evidence of product formulation documents is kept by the business owner confidential.

#### 3.3.8. Production

Production is carried out at facilities that meet the criteria, halal, and free from haram substances and najis (ritually unclean) materials. This requirement applies to self-owned production facilities and other parties' facilities if subcontracting production. Procedures must guarantee that the halal inspector approves all materials for production. Producers must meet product quality standards assessed using materials, equipment, production processes, and labor in the production process [50]. Production activities in the Happy Cake, Canggi Fully, and MacCheese bakery are in one kitchen. Before carrying out production, employees must wear closed clothing, headgear, and gloves when processing materials.

**Figure 4:**
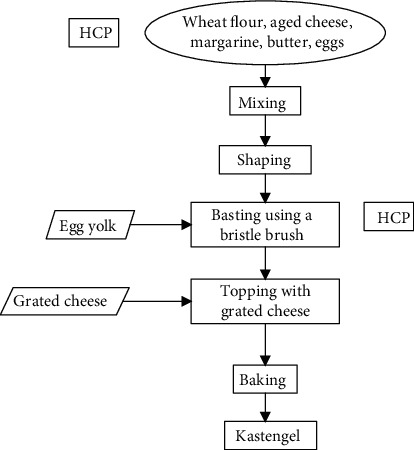
The production process of kastengel and its HCP.

Production in these bakery industries is carried out after receiving a customer order. The processing of one bakery type is different from another bakery. The ingredients of every bakery producers are prepared according to their recipe portion. The material must not be produced from the facilities for process products containing pigs and their derivatives [51]. The following is an example of the production process, along with HCP kastengel products in Figure 4.

Kastengel production has two HCP. There are Edam cheese and a brush. Edam cheese is cheeses from Dutch that are traditionally round, bright yellow, and wrapped in paraffin and red plasticine. Cheese is coagulated using the rennet enzyme. To improve the appearance of cheese is to added flavor and emulsifier. The rennet enzyme is a type of protein-breaking enzyme. This enzyme is produced from young mammals' stomach glands, especially calves [24].

The flavor is added to give aroma or taste. The flavor provides a savory and delicious taste known as flavor enhancers. Flavors give a fragrant flavor, such as fruit flavor, called flavor fragrance [24]. The flavor is a complicated ingredient from vegetable, animal, and synthetic, so every material must be observed. For example, vanilla flavor is produced by various methods involving microbes [52, 53]. The origin of the microbe must be traced.

The emulsifier forms stabilize emulsions and help bond oil with water. Most emulsifiers are from fat-derived compounds such as monoglycerides and diglycerides. Making emulsifiers involves a source of fat, fatty acids, and glycerol [39]. Fat from animals, the halal status depends on the type of animal and its slaughter. Brush bristles from bristle or animal hair need to be aware of halal. Brushes from animal hair include HCP. If it is made of haram animal hair such as pig, it should not be used because it is haram and unclean, dry, or wet [41].

#### 3.3.9. Washing of Production Facilities and Equipment

Washing facilities in 3 bakery businesses are carried out after production using flowing water. Washing in small enterprises is closely related to the design of production and the equipment used [54]. In these small bakery businesses, the washing process is simple. A washing uses liquid dishwashing soap for all types of tools. The fishy smell is washed using soap and hot water. Washing using flowing water is better than soaking [55]. Washing using running water removes all dissolved impurities without recontamination of the equipment. Some production facilities such as ovens, pans, mixers, stirring machines, and coolers (refrigerators and freshers) are cleaned before using every morning. The pan is always smeared with oil/butter then heated in the oven to clean. The mixer is cleansed of the remaining dough that is sticking with oil. The bread cooling rack is cleaned of bread crumbs and dust that clings to it using a clean cloth. All production equipment must be properly sanitized [56].

#### 3.3.10. Storage and Handling of Materials and Products

Material storage is a method of organizing, storing, and maintaining food ingredients (dry and wet) in the appropriate material storage area. Malaysian Standard has set logistic standards through MS 2400: 2010 [57]. Therefore, food storage is aimed at ensuring that ingredients are not easily damaged and lose nutrition and at guaranteeing their halalness. Material storage and handling at three bakery businesses have the same procedure. Meat ingredients are stored in the freezer to avoid microbial contamination. Food safety is very supportive and a prerequisite for the halal production process program [58]. Hygiene and sanitation are also prerequisites in the halal production process program, including premises for storage [59]. The storage and handling material area must be maintained hygienically, arranged adequately to avoid pests, leakages, messy orders, and negligent placement [60]. Fresh ingredients such as vegetables, fruit, and various pasta are stored in the refrigerator to maintain freshness. Dry ingredients such as flour and nuts are stored in wood cabinets and dry aluminum for durability. Dry matter can be stored at room temperature if it is appropriately packed. This storage model fulfills the need to ensure the halalness of the material.

#### 3.3.11. Transportation

Transport procedures must be observed to ensure halal products' contamination by haram (unlawful) or najis (unclean Islamic ritually) materials during transportation [43]. Many factors influence halal transportation and logistics [61]. Shipping in the Happy Cake bakery business is done manually by employees, while in Canggi Fully and MacCheese, the business owner does it. Transportation used car and motorcycle to their destination market. Likewise, when suppliers send materials to bakery production place. In the Happy Cake supplier bakery business, employees bring materials to the storage warehouse. The material delivered by the supplier is ensured packed tightly and well to ensure product quality. In addition to transportation, transportation must be free of unclean or illegal materials. Transporting materials from suppliers must be clean from haram or najis substances and not transport haram material, animals, or humans. The halal supply chain in production is essential to halal product assurance [62].

### 3.4. Internal Audit of Critical Activity Procedures

**Figure 5:**
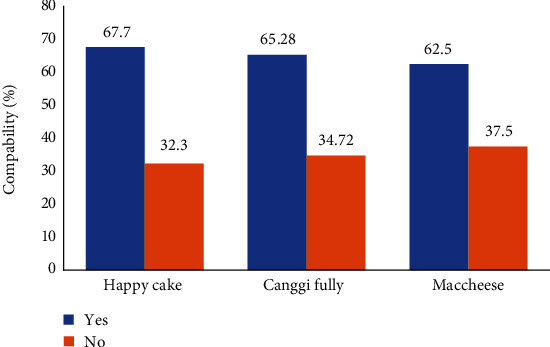
Internal audit results of critical activity procedures. Yes: under the state of the bakery business; No: not according to the state of the bakery business.

The internal audit assesses the suitability of documents and HAS's application in each bakery business with the halal examining body's requirements. Internal audits of all critical activity procedures are conducted, from material selection to transportation. Internal audits are conducted at least once every six months or more (if needed)—the internal audit results are in Figure 5.

Figure 5 shows the results of an internal audit for the written procedure for critical activities. In Happy Cake bakery, 67.7% answered yes, and 32.3% responded no; in Canggi Fully, 65.2% answered yes, and 34.7% answered no, while in MacCheese business, 62.5% answered yes, and 37.5% answered no. There is a mismatch between the respondent's answers and the actual situation in business. Internal audit results of Happy Cake, Canggi Fully, and MacCheese bakeries are almost the same. These three businesses are small-scale that are just starting to paying attention to HCP materials and halal production processes. This discrepancy also occurs in food safety in developing countries [63]. These conditions need to be improved, especially the inspection of materials and arranging documents to prove critical activities' suitability.

### 3.5. Handling of Products That Do Not Meet the Criteria

Based on the material search results, there is no brand and no halal label, including HCP material. The halal substance is doubtful, given a substitute alternative. Three bakery businesses conduct register halal certification at the research, and this material replacement must be done. The choice of substitute materials is based on the ease of obtaining materials at the market, price, and taste and quality materials for production. Also, there is a halal certificate on the product. Alternative substitute materials can be shown in Table 5.

The halal certification of three businesses requires replacing non-halal-certified material with clearly halal material. In Indonesia, old cheese/Edam cheese has not been certified halal replaced by imported aged cheese, which has been certified halal, namely, the Anchor brand. Canned donut sugar and canned cherries are not halal, replaced by Semut brand of donut sugar, and halal certified Wilmond brands replace canned cherries. Essence (rhum) is replaced by Toffico zero black forest food flavorers with a taste and aroma like rhum but does not contain alcohol.

The flavor is made synthetically chemically with content: water, sugar, propylene glycol, and identical natural flavor. The flavor is often made from fermented glycerin [64]. The propylene glycol is an organic compound with a hydroxyl group with C_3_H_6_ (OH)_2_. It is a thick, colorless, odorless, slightly sweet, hygroscopic, and water-soluble liquid [24]. Propylene glycol is used as a glycerin substitute. Glycerin is a thick liquid that tastes sweet and is trihydroxy alcohol. Glycerin can be obtained from depositing fats or oils (animal or vegetable) and sugar fermentation. Oil from animals must be ascertained from halal animals and slaughtered according to Islamic rules. This material is one of the HCPs from glycerin as a flavor ingredient [39]. Fermentation uses media which also needs to be guaranteed its halalness. This synthetic flavor has the advantage of removing fishy odors and aromas that are resistant to baking and has a halal certificate to be used for production. The brush is replaced with a brush from plastic/silicone that has been certified halal.

### 3.6. Implications of Material Substitution for the HAS Implementation

Alternative guaranteed halal ingredients have been given to three small-scale bakeries to meet the halal certification requirements. Bread making has HCP in mixing with other ingredients [49]. Therefore, materials that are not yet halal must be replaced with clearly halal materials. For example, the old Victorian cheese worth IDR 135,000/800 gr replaced the Anchor brand worth IDR 175,000/800 gr. The substitution of materials affects production costs, product quality, prices, and profits. Production and operating costs will determine business profits [65]. Product quality is affected by materials and processes [66]. The ingredient substitution can impact product quality. Therefore, material replacement needs to be examined so as not to degrade the product quality. Material selection procedures and material choice are essential. Changing materials should recognize the ingredients' characteristics, competitive price, and available on the nearest market [67]. Material substitution in three bakeries has been completed, and halal certification submission is carried out on the Halal Certification Body in Indonesia. After an audit of the halal auditing agency and improvements to the field audit records, the three small-scale bakeries have been certified halal. The halal production process affects the sustainability of food SMEs [50].

## 4. Conclusions

Based on the decision tree Halal Control Point (HCP) mapping in three small-scale bakeries, there are non-HCP (halal) materials, HCP, and no haram (unlawful) material. The Happy Cake bakery has 75% non-HCP (halal) materials and 25% HCP or uncertified halal materials from 80 bakery ingredients. Canggi Fully's bakery has 83.3% halal-certified materials and 16.6% non-halal-certified materials from 26 components. Despite this, the MacCheese bakery has 79% halal materials and 21% non-halal-certified materials from 43 ingredients. The decision tree is beneficial to trace the halal status of materials. Uncertified halal ingredients, such as donut sugar, canned cherries, essence (rhum), and old cheese, must be replaced with the clearly halal material. Halal material choices affect production costs, product quality, and profit. A commitment of small-scale bakery business actors to choose materials determines the speed of compliance with halal certificate requirements.

The findings of this study have several practical implications; namely, material changes in production costs, product quality, and operating profit have not been calculated in detail to be investigated next time. Raw materials are selected as halal-certified because of a requirement for the registration of halal certification. Written documents need to be made to guarantee the halalness of the product. Facilities and equipment in small-scale bakeries need to be arranged to avoid the risk of cross-mixing of haram/najis materials in the product.

## Figures and Tables

**Table 1:** List of bakery products.

No.	Bakery name	Bakery type	Product example
1	Happy Cake	Bulger, green tea cake, pie pot, pizza, pinky potato, fruit puff, red velvet puff, ragout choux pastry, klapertart (coconut cake), chocolate flush pie, eclair, chicken potato pie, floss croissant, almond chocolate croissant, mushroom croissant, strawberry croissant, blueberry croissant, oreo chocolate croissant, almond croissant, strawberry patella, blueberry patella, chocolates patella, oreo chocolate patella, almonds patella, nastar (pineapple tart), putri salju (powdered sugar coating cookie), kastengel (cheese cookie), and chocolate drop chips	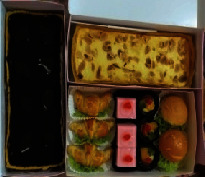
2	Canggi Fully	Wet chocolate choux pastry, wet vanilla choux pastry, brownies pie, moringa crackers, brownies crackers, nastar, putri salju, and kastengel	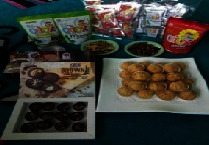
3	MacCheese	Mac and cheese, marble cake, cheese roll, banana bolen, Singapore's pastel (karipap), nastar, papizza, and kastengel	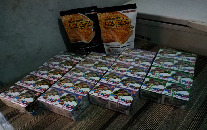

**Table 2:** HCP identification data of animal materials in bakery based on decision trees.

Bakery name	Material	Question						Result	Potential ingredients/processes are not yet halal
		A	A1	B	B1	B2	B3		
Happy Cake	Beef floss	—	—	√	√	√	√	HCP A2	Slaughter process, MSG
	Milk powder	√	√	—	—	—	—	HCP A1	Lactose, stabilizer, and emulsifier
	Shrimp	√	x	—	—	—	—	Non-HCP (A)	—

Canggi Fully	Whip cream	√	√	—	—	—	—	HCP A1	Milk, emulsifier, and flavor
	Butter	√	√	—	—	—	—	HCP A1	Milk, fat, and emulsifier
	Chicken egg	√	x	—	—	—	—	Non-HCP (A)	—

MacCheese	Chicken meat	—	—	√	√	√	√	HCP A2	Slaughter process
	Cheddar cheese	√	√	—	—	—	—	HCP A1	Enzymes in the coagulation process of milk, emulsifier, and flavor
	Flavoring	—	—	√	√	√	√	HCP A2	MSG

HCP A (number). HCP: Halal Control Points; A: animal material code; A1: group of ingredients (milk, eggs, and fish) with processing; A2: group of ingredients (meat and derivatives) without processing that does not have a halal certificate; A3: groups of ingredients (meat and their derivatives) by processing; Non-HCP (A): material not critical for animal material.

**Table 3:** HCP identification of vegetable material based on the decision tree.

Bakery name	Material	Questions				Result	Potential ingredients/processes are not yet halal
		C	C1	C2	C3		
Happy Cake	Margarine	√	x	—	√	HCP B2	Antioxidant, colorant, flavor, emulsifier
	Strawberry jam	√	x	—	√	HCP B2	Flavors, sugar, preservatives, colorants
	Wheat flour	√	x	—	√	HCP B2	Vitamin A and L-cysteine to enhance wheat flour properties

Canggi Fully	Pineapple	x	—	—	—	Non-HCP	—
	Chocolate powder	√	x	—	√	HCP B2	Sugar, milk, lecithin, chocolate flavor, and emulsifier
	Fine granulated sugar	√	x	—	√	HCP B2	Activated carbon and resin in the refining process

MacCheese	Instant curry	√	x	—	√	HCP B2	Sugar, preservatives, and thickeners
	Potato	x	—	—	—	Non-HCP	—
	Tomato sauce	√	x	—	√	HCP B2	Sugar, flavor, oleoresin, and MSG

HCP B (number). HCP: Halal Control Points; C: vegetable material processing-question code; C1: group of ingredients with the fermentation process (microbial cultivation); C2: group of ingredients with the khamr (alcohol) fermentation process; C3: groups of materials with processing and addition; Non-HCP (B): material not critical for vegetable matter.

**(a):** 

No.	Material	Place of purchasing material
	Happy Cake	
1	Chicken meat	Wahana slaughtering in Batu, East Java
2	Puff pastry sheets	Supplier in Malang region
3	Grafe, kiwi fruit, and canned fruit (lemon and orange)	Lai-lai, Malang City
4	Eggs, onions, lettuce, purple sweet potatoes, carrots, potatoes, ground pepper, ground nutmeg, peppers, pineapples, prawns, shallots, garlic, celery, cooking oil, and young coconut	Mergan traditional market
5	Wheat flour, cornstarch, yeast, sugar, salt, margarine, cheese, chicken meat (patty), mayonnaise, tomato sauce, milk, oregano, sausages, pasta, canned cherries, sprinkles, instant coconut milk, cinnamon powder, walnuts, raisins, butter, cream cheese, jam, Nutri jelly, chocolate, food coloring, flavor, choco chips, shredded beef, instant broth, cashews, and almonds	Bakery material shop, Primarasa Kyai Tamin street, Sukoharjo, Klojen Malang

**(b):** 

	Canggi Fully	
1	Eggs, moringa leaves, and pineapple	Big Market, Malang City
2	Sugar, cornstarch, wheat flour, cocoa powder, margarine, choco chips, milk, white chocolate, almonds, chopped nuts, cheese, whip cream, salt	Bakery material shop diva, Jalan Kyai Tamin street, Sukoharjo, Klojen, Malang City

**(c):** 

	MacCheese	
1	Chicken meat and smoke beef	Superindo supermarket
2	Pineapple, garlic, potatoes, bananas, carrots, eggs	Sawojajar traditional market
3	Wheat flour, cheese, margarine, tomato sauce, milk, yeast, sugar, gram, oregano, milk powder, butter, Danish pastry, chocolate, emulsifier, kare or curry seasoning, and ground nutmeg	Bakery material shop Amelia, Danau Bratan Raya street, Malang City

**Table 5:** Alternative replacement of materials and tools.

No.	Material	Brand/supplier	Alternative material
1	Sugar donut	Prima Fine Sugar/Primarasa Malang	Sugar donut Claris/Prigent
			Sugar donut Semut/CV. Usaha Baru^∗^
			Sugar donut ALCO/PT Karya Anugerah Jaya
2	Edam/mature cheese	Victoria Edam/Primarasa Malang	Edam cheese: Anchor/Fonterra Cooperative Group Ltd.^∗^
			Edam cheese: cheese/PT. Dairygold Indonesia
			Edam cheese: old Gouda cheese/Molfino HNOS S.A
			Edam cheese: Valley shredded/PT. Pacific Lacto Jaya
3	Canning cherry	Royal Willamette Cherries	Wilmond Canned Pitted Cherries/Zhejiang Ju Zhen Yuan Foodstuffs Co., Ltd.^∗^
4	Essence (rhum)	Butterfly/Diva Malang	My vla/PT. Forisa Nusa Persada
			Toffieco Zero Black Forest/Pillarose^∗^
5	Brush		Brush from plastic/silicon has halal-certified^∗^

^∗^Alternative selected bakery business.

## Data Availability

The authors declare that they have all the necessary data and are available where appropriate or requested by the editor.
